# Exploring the genetic frontier: Unraveling ANRIL, PAI-1, and HNF1α in stroke progression

**DOI:** 10.1371/journal.pone.0332252

**Published:** 2025-12-11

**Authors:** Abdullah Hamadi, Rashid Mir, Osama M. Al-Amer, Mohammed Alasseiri, Gasim Dobie, Imadeldin Elfaki, Waseem AlZamzami, Sael Alatawi, Atif Abdulwahab A. Oyouni, Mohammad A. Alanazi, Saravanan Muthupandian

**Affiliations:** 1 Faculty of Applied Medical Sciences, Department of Medical Laboratory Technology, University of Tabuk, Tabuk, Saudi Arabia; 2 Prince Fahad Bin Sultan Chair for Biomedical Research, University of Tabuk, Tabuk, Saudi Arabia; 3 Faculty of Applied Medical Sciences, Department of Medical Laboratory Technology, Jazan University, Jazan, Saudi Arabia; 4 Department of Biochemistry, Faculty of Science, University of Tabuk, Tabuk, Saudia Arabia; 5 Department of Biology, Faculty of Science, University of Tabuk, Tabuk, Saudia Arabia; PLOS: Public Library of Science, UNITED STATES OF AMERICA

## Abstract

**Background:**

Ischemic stroke poses a notable global public health challenge, with the Kingdom of Saudi Arabia (KSA) being no exception. This multifaceted condition is affected by a combination of factors, including hypertension, diabetes, and genetic influences. The purpose of the present study was to examine the linkage of long noncoding RNAs (such as ANRIL), plasminogen activator inhibitor-1 (PAI-1), and hepatocyte nuclear factor 1 alpha (HNF1α) gene variations with stroke. Leveraging a substantial cohort comprising both stroke patients and healthy individuals from KSA, our research revealed numerous uncommon genetic variations linked to an increased predisposition to stroke. This insight enhances our comprehension of stroke’s genetic underpinnings and can be invaluable in formulating preventive measures, not only in KSA but also on a global scale.

**Methods:**

In this study we included 100 stroke patients and 100–120 healthy controls from Saudi population. We utilized the amplification refractory mutation system-PCR to genotype the chromosome 9p21 locus, the long noncoding RNA-ANRIL (lncRNA-ANRIL), Hepatocyte Nuclear Factor 1 alpha (HNF1α-A > C [p.I27L]) gene, and the plasminogen activator inhibitor-1 (PAI-1) gene to investigate the association of these gene variations with a stroke. Additionally, WES was performed for 10 stroke patients using the Illumina NovaSeq 6000 platform.

**Results:**

Our investigation revealed significant associations between stroke patients and healthy controls concerning polymorphic variants of lncRNA-ANRIL (A > C), ANRIL (G > A), HNF1α-A > C, and PAI-1-4G > 5G genes (p < 0.05). Notably, the ANRIL rs1333048-GA genotype exhibited a strong connection with stroke susceptibility in both codominant (OR 2.29, RR 1.54, P < 0.007) and dominant (OR 1.83, RR 1.35, P < 0.034) models, while an overdominant inheritance model demonstrated a protective correlation (OR 0.42, RR 0.64, p < 0.004). Similarly, the ANRIL rs10757278-GG genotype was significantly associated with stroke susceptibility in the codominant (OR 2.80, RR 1.66, P < 0.004) and recessive (OR 3.14, RR 1.62, P < 0.0003) models, with the G allele also displaying a significant association. The HNF1α-TT genotype strongly correlated with stroke risk in the codominant (OR 18.36, RR 9.96, P < 0.048) and recessive (OR 22.14, RR 10.18, P < 0.034) models, with the HNF1α-T allele following a similar trend. The PAI-1-4G-5G genotype was also linked to stroke predisposition (OR 2.09, RR 1.34, P < 0.034) and an increased risk in the dominant model (OR 2.27, RR 1.43, P < 0.006). Furthermore, our study identified several novel and common gene variants in stroke patients through WES, including COL4A2, PSEN2, NOTCH3, and RNF2.

**Conclusion:**

Our findings underscore the significant role of genetic determinants in chromosome 9p21, the lncRNA-ANRIL, HNF1α-A > C (p.I27L), and PAI-1-4G > 5G genes in elevating the risk of stroke. Additionally, we report low, novel, and intermediate-genetic-risk variants in COL4A2, PSEN2, NOTCH3, and RNF2 through WES, emphasizing the need for further investigation in larger cohort studies.

## Introduction

Stroke is a significant global cerebrovascular disorder, claiming around 6 million lives annually or more than 10% of all global fatalities and imposing a substantial economic burden on healthcare systems [1]. In 2019, there were a total of 7.63 million cases of strokes, with ischemic strokes (ISs) comprising 62.4% of them [2]. The World Health Organization (WHO) defines ISs as episodes of neurological dysfunction caused by localized cerebral, spinal, or retinal infarction. Unfortunately, survivors of IS frequently experience significant disability [3]. The incident stroke mean age was estimated as 74.3 years (SD: 13.6), with men showed incident stroke at a younger age in comparison to women (71.4 vs. 76.9 years) [4]. The Kingdom of Saudi Arabia (KSA) is witnessing a rise in stroke-related mortality rates, underscoring the urgent need for comprehensive prevention strategies [5]. In addition to well-known risk factors like metabolic syndrome, characterized by abdominal obesity, hypertension, type 2 diabetes, smoking, physical inactivity, psychosocial stress, depression, hypertension, and cardiac diseases [6], genetic predisposition plays a significant role in stroke causes [7–9]. In KSA and Arab countries, there is a higer the rate of consanguineous marriage in comparison to the Western European and Asian countries [10]. This higer the rate of consanguineous marriage is associated with increasing risk of genetic disorders, including congenital heart diseases, stroke, diabetes mellitus, kidney diseases, and rare blood diseases [10,11]. Recent studies have revealed the substantial impact of genetics on IS, especially in early-onset cases. Various stroke classification systems have emerged, with polygenic disorders accounting for about 38% of all IS cases. These genetic insights offer promising avenues for personalized stroke prevention and innovative treatments, emphasizing the importance of continued research and clinical efforts in stroke management.

Non-protein coding areas make up at least 98% of the human genome. Eighty percent of the 98% areas are transcribed to RNAs. These areas’ transcripts have long been thought of as transcriptional “noise” [12]. Lately, the manifestation, operation, and processes of These non-coding RNAs have garnered a lot of interest. Non-coding RNAs are separated into two categories based on length: large (>200 nt) RNAs (ribosomal RNAs and lncRNAs) and tiny (<200 nt) RNAs (microRNAs and transfer RNAs) [13]. The fact that ncRNAs are expressed differently in different tissues and at different stages of development shows that they play a big role in both healthy and sick processes. Several findings point to ncRNAs as a possible cause of stroke [14].

Noncoding RNAs (ncRNAs), including microRNA (miRNA), long noncoding RNA (lncRNA), circular RNA (circRNA), and PIWI-interacting RNA (piRNA), play crucial roles in stroke [15]. Recently, elevated expression of antisense non-coding RNA in the INK4 locus (ANRIL), with alleles on chromosome 9p21 was found to be associated with cardiovascular diseases [16], a major risk factor for stroke [17]. More presicely, ANRIL SNP rs1333048, was shown to substantially impact the risk of cardiovascular disease [18]. While its heterozygous carriers exhibited an intermediate risk level, homozygous carriers had a heightened risk of developing coronary atherosclerosis [18]. The genetic rs10757278 polymorphism on human chromosome 9p21 has been suggested to influence lipid metabolic syndrome [8], particularly affecting cholesterol and low-density lipoprotein (LDL) levels, thus impacting the risk of cardiovascular disease [19], and an increased risk of stroke [20,21]. Hu X. et al. have highlighted a robust association between the rs10757278 variant and environmental factors, including smoking, which further modulate the risk of stroke [20,21]. Recent findings indicate a higher prevalence of the rs10757278 variant among individuals of European and Asian descent exhibit an increased risk of cardiovascular disease [22], suggesting it a genetic indicator [23]. Similar to ANRIL, rs1169288 SNP of Hepatocyte Nuclear Factor 1 alpha (HNF1α) has been observed in young diabetic patients [15], and association with insulin resistance [24]. While the role of HNF1α has been observed in hypertension, dyslipidemia, and genetic variations [25], it remains unclear whether HNF1α rs1169288 SNP is involved in metabolic syndrome, cardiovascular disease [26], and stroke [26].

Mutations mostly 4G/5G guanine nucleotides insertion/deletion polymorphism at position 675 of the Plasminogen activator inhibitor-1 (PAI-1) promoter [27,28], leads in its increased levels and result in decreased plasma fibrinolytic activity, a phenomenon observed in metabolic syndrome [29], cardiovascular disease and myocardial ischemia [30,31]. In addition, the thrombotic phenotype in individuals with prothrombotic conditions is correlated with the PAI-1 4G/5G gene polymorphism, which may impact the prognosis of patients with cardiovascular disease [32]. Notably, El-Tarras et al. identified a higher prevalence and allele frequency of this polymorphism in high-altitude cardiovascular patients compared to those residing at sea level, suggesting a relationship between hypoxia, the polymorphism, and the geographic altitude of patients in Saudi Arabia [33]. Though PAI-1 genotype has been shown to influences the likelihood of future stroke [34], role of 4G/4G genotype in the risk of stroke is still debatable [35]. Here in, we investigated the complex interplay between genetic factors, lncRNAs, and various genetic polymorphisms that underscores the intricate web of influences in predisposing indidulas to stroke in the KSA.

## Methodology

### Study population

Following the ethical approval granted by the University of Tabuk’s Ethics Committee (UT-111-19-2020; date of approval 15/05/2020) following the principles of the Helsinki Declaration, written informed consent was obtained from all participants before sample collection. This genetically homogeneous study exclusively included Saudi Arabs and excluded non-Saudi Arabs, non-Arabs, and recently naturalized Arabs. The study comprised 220 participants, 105 individuals diagnosed with stroke, and 115 healthy controls. Samples were collected from various sources, including King Khaled Hospital, King Salman Military Hospital-Tabuk, and during routine medical checkups from 01/09/2020 till 31/03/2021.

### Diagnostic markers for stroke

Physical examinations and analysis of brain scan images. Common stroke mimics include seizures, somatoform disorders (conversion), migraine headaches, and hypoglycemia. Physical exams include blood pressure readings and cardiac auscultation. Neurological examinations assess the impact of a potential stroke on the nervous system. Diagnostic tests may include hematologic testing, magnetic resonance imaging (MRI), carotid ultrasound, computed tomography (CT) scans, cerebral angiograms, and echocardiograms. Data were collected from tests conducted in the hospital and from a standardized questionnaire that included demographic information such as systemic hypertension (blood pressure readings above 140/90 mmHg or past antihypertensive medication usage, age and gender. Samples were collected from various sources, including King Khaled Hospital, King Salman Military Hospital-Tabuk, and during routine medical checkups during the period during the period from January 2021 to july 2023.

### Specimen and data collection from the stroke patients

Blood samples from stroke patients, approximately 3 mL in volume, were collected using EDTA or Lavender top tubes. Healthy control samples were obtained during routine blood draws to minimize additional phlebotomy. All blood samples were promptly stored at temperatures ranging from −20 °C to −30°C. Data collection involved the use of a standardized questionnaire that encompassed demographic details such as age and gender, as well as cerebrovascular risk factors, which included systemic hypertension (defined as having two outpatient blood pressure measurements above 140/90 mmHg or prior use of antihypertensive medication), dyslipidemia (defined as total serum cholesterol > 200 mg/dl, LDL > 100 mg/dl, HDL < 50 mg/dl, triglycerides > 150 mg/dl, or medication use, e.g., statins or lipid-lowering agents), diabetes mellitus (as per previous diagnosis or current medication use), any history of atrial fibrillation from prior monitoring, and the current smoking status.

### Genomic DNA extraction and genotyping

Genomic DNA was extracted from patient and healthy group samples using the Qiagen DNeasy Blood Kit (Hilden, Germany) following the manufacturer’s instructions. Subsequently, the DNA samples were dissolved in nuclease-free water and stored at 4°C. The quality and integrity of the extracted DNA were assessed using the NanoDrop™ spectrophotometer (Thermo Scientific, Waltham, Massachusetts, USA). The genotypes of ANRIL- rs1333048 A > C, ANRIL- rs10757278 G > A, HNF1α- rs1169288 A > C (I27L), and PAI-14G/5G gene variations were determined using ARMS PCR (Amplification Refractory Mutation System PCR) and Allele-specific PCR methods [36]. Specific primers for the genotyping of these variations were designed using the Primer3 software, and details of these primers can be found in Table 1.

**Table 1:** Details of primers used for genotyping.

**ARMS primers for lncRNA ANRIL - rs1333048 A > C**			
LNCR048-F0	TTGCCTGATTACCAATTTTATATGTTA	382 bp	45 ^0^C
LNCR048-R0	TCAACTGATGATGATATGGTTAGTATG		
LNCR048-FI-A	TTAATGCTATTTTGAGGAGATGTCTA	185 bp	
LNCR048-RI-C	TTTTATCAATATTTCAATAATTCGACACTG	253 bp	
**ARMS primers for lncRNA ANRIL rs10757278G>A**			
LNCR278F0	5′-GGCATTAAGAAATGGATGGGTAGACAAAA-3′	443 bp	54 ^0^C
LNCR278R0	5′-GCTGTTCTCAATTAGCCAGGACTACCTCT-3′		
LNCR278FI-A	5′-AAGTCAGGGTGTGGTCATTACGGGAA-3′	263 bp	
LNCR278RI-G	5′-CTCAGTCTTGATTCTGCATCGCTTCC-3′	234 bp	
**ARMS primers for hepatocyte nuclear factor-1 alpha *HNF1α* (rs1169288) A > C .I27L**			
HNF1A-Fo	5′-GTGCCCACAGGGCTTGGCTAG-3′	387 bp	62 ^0^C
HNF1A-Ro	5′-CCATCGTCGTCCGTCTCGTCCTCG-3′		
HNF1A-FI-C	5′-GGGCTGAGCAAAGAGGCACCG-3′	176 bp	
HNF1A-RI-A	5′-CCCGGCTCACCCAGTGCCTGAAT-3′	257 bp	
**ARMS primers for plasminogen activator inhibitor-1 − 675 4G/5G promoter polymorphism**			
PAI-1 Fo	5′-AAGCTTTTACCATGGTAACCCCTGGT-3′	256 bp	55 ^0^C
PAI-1 Ro	5′-TGCAGCCAGCCACGTGATTGTCTAG-3′		
PAI-1 4G FI-4G	5′-AGAGTCTGGACACGTGGGGA-3′	138 bp	
PAI-1 5G RI-5G	5′-AGAGTCTGGACACGTGGGGG-3′	138 bp	

### Gel electrophoresis

The ANRIL- rs1333048 A > C genotyping revealed a 382 bp control band produced by amplifying primers FO and RO to assess DNA quality and quantity. A 185 bp band (A allele) resulted from the amplification of primers FI and RO, while a 253 bp band (C allele) was produced by the amplification of primers FO and RI (Table 1). For ANRIL- rs10757278G>A, a 443 bp control band was generated by amplifying primers FO and RO for DNA quality and quantity assessment. The A allele resulted in a 263 bp band using primers FI and RO, and the G allele produced a 234 bp band. Regarding the Hepatocyte nuclear factor-1 alpha (HNF1Α) rs1169288 A > C (I27L), a 387 bp control band was obtained through the amplification of primers FO and RO for DNA quality and quantity assessment. The C allele produced a 176 bp band using primers FI and RO, while the A allele generated a 257 bp band. For the PAI-1 gene (675, 4G/5G) (rs17998894 4G/5G), the −675 4G/5G polymorphism was detected using ARMS-PCR. A 256 bp control band was produced by amplifying primers FO and RO for DNA quality and quantity assessment. A 138 bp band was generated when the forward 4G allele-specific primer or 5G allele-specific primer combined with the reverse of a common downstream primer.

### Whole exome sequencing

Library preparation was carried out for WES as per the Twist 2.0 Exome kit’s instruction manual, and sequencing was performed following the Illumina NovaSeq 6000 platform’s user guide. Quality control of sequencing reads was performed using FastQC v0.11.9, and raw reads were filtered to remove low-quality bases and sequencing adapters using TrimGalore v0.6.6. After mapping the high-quality reads to the hg38 human reference genome, variation calling (SNVs, tiny InDels) was conducted using the GATK (v4.2.4.1) best practice pipeline and the haplotype caller. Various databases and technologies were employed to annotate the identified variants, with gene-related variations described using the RefSeq database. OMIM and ClinVar databases were used to assess the disease associations of the variations. To filter out common variations and polymorphisms, population frequency data from 1000 Genomes, ExAC, GnomAD exome, GnomAD genome, and ESP were utilized. The effects of coding non-synonymous SNVs on protein structure and function were estimated using PolyPhen-2 and SIFT scores. Additionally, in-silico variant effect predictions were performed using multiple prediction algorithms. Subsequently, all variants were classified as pathogenic, potentially pathogenic, or variants of unknown significance based on ACMG standards [37].

## Results

### Demographic features of stroke patients

A comparative analysis was conducted between the healthy control group (mean age = 35) and the stroke patients (mean age = 58), focusing on various biochemical parameters. The results revealed statistically significant differences in most of the analyzed biochemical markers in stroke patients. Table 2 illustrates substantial variations between the two groups’ serum lipid profile and blood glucose status. Stroke patients exhibited elevated blood sugar levels (fasting), glycated hemoglobin, cholesterol, LDL, VLDL, and triglycerides compared to the healthy controls. However, no significant differences were observed in platelet count and liver enzyme levels (ALT/AST). Additionally, the study explored the significance of gene variations among different allelic forms and statistically correlated them with the gender and age of stroke patients.

**Table 2:** Comparative clinical characteristics of the study population.

Characteristic	Controls^a^	Patients	*P* ^b^
Age	35.63 ± 12.81	58.44 ± 9.43	<0.001
Blood sugar fasting	98.97 ± 5.42	101.88 ± 5.41	<0.001
HbA1c	5.097 ± 0.402	6.027 ± 0.314	<0.001
Triglyceride	127.08 ± 9.42	165.9 ± 32.2	<0.001
Cholesterol	114.37 ± 8.20	171.2 ± 50.1	<0.001
HDL	48.1 ± 11.8	25.44 ± 3.57	<0.001
LDL	115.19 ± 9.65	148.4 ± 31.8	<0.001
VLDL	. 27.81 ± 5.40	43.3 ± 13.8	<0.001
Platelet count	235.9 ± 72.6	250.5 ± 84.3	0.171
AST	25.1 ± 10.3	27.7 ± 16.1	0.158
ALT	25 ± 10.2	30.7 ± 24.6	0.580

^a^student’s t-test for continuous variables (variables with normal distribution),

^b^Values are presented as mean ± standard deviation.

### Distribution of genotypes and alleles of ANRIL, HNF1α and PAI-1 genes in stroke patients

Our study uncovered significant disparities in the distribution of genotypes for several genetic variants in stroke patients compared to healthy controls. A significant difference was noted in the case of ANRIL-rs1333048 A > C genotypes (p ≤ 0.015). Among stroke patients, the genotypes included CC (40%), CA (50%), and AA (10%), while healthy controls exhibited CC (55%), CA (30%), and AA (15%) genotypes (Table 3). Furthermore, the A allele was more prevalent among stroke patients (0.35) than in healthy controls (0.30). Likewise, a significant difference was observed for lncRNA-ANRIL rs10757278 G > A genotypes (p < 0.0049). In stroke patients, the genotypes were distributed as AA (24%), GA (35%), and GG (41%), whereas in healthy controls, the distribution was AA (34.26%), GA (45%), and TT (20.83%) (Table 3). Additionally, stroke patients had a higher prevalence of the G allele compared to healthy controls, with respective frequencies of 0.58 and 0.43. Regarding HNF1α (rs1169288) G > T (I27L) genotypes, stroke patients exhibited GG (37%), GT (55%), and TT (8%), while healthy controls had GG (16.66%), GA (66.66%), and TT (0%) genotypes (p < 0.004). Stroke patients also showed a slightly higher frequency of the T allele than healthy controls, with frequencies of 0.35 and 0.33, respectively. In the case of PAI-1 (rs1799889) 4G > 5G promoter genotypes, the distribution significantly differed between stroke patients and healthy controls (p < 0.014). Among stroke patients, genotypes included 5G (24%), 5G/4G (45%), and 4G (31%), while healthy controls had 5G (41.81%), 4G/5G (39%), and 4G (19%) genotypes (Table 3). Additionally, it was observed that stroke patients had a higher frequency of the 4G allele compared to healthy controls, with respective frequencies of 0.53 and 0.38 (Table 3).

**Table 3:** Association of ANRILrs1333048 A > C, rs10757278 G > A, HNF1α rs1169288 G > T (I27L) and PAI-1- 4G > 5G rs1799889 4G > 5G genotypes in stroke patients and controls.

**Association of long noncoding RNA -ANRILrs1333048 (A/C) with stroke patients**									
**Subjects**	N=	CC (%)	AC	AA	C	A	DF	X2	P value
**Cases**	100	40 (40%)	50 (50%)	10 (10%)	0.65	0.35	2	8.37	0.015
**Controls**	100	55 (55%)	30 (30%)	15 (15%)	0.70	0.30			
**Association of long noncoding RNA-ANRIL rs10757278 A > G with stroke patients**									
**Subjects**	N=	AA	AG	GG	Df	X^2^	A	G	
**Cases**	100	24 (24%)	35 (35%)	41 (41%)	2	10.65	0.42	0.58	0.0049
**Controls**	120	41 (34.26%)	54 (45%)	25 (20.83%)			0.57	0.43	
**Association of Hepatocyte nuclear factor-1 alpha *HNF1α* (rs1169288) G > T (I27L) with stroke patients**									
**Subjects**	N=	LL) GG	IL (GT)	II (TT)	Df	X^2^	G	T	p- value
**Cases**	100	37 (37%)	55 (55%)	8 (8%)	2	11.2	0.65	0.35	0.004
**Controls**	120	40 (16.66%)	80 (66.66%)	0 (0%)			0.67	0.33	
**Association of Plasminogen activator inhibitor-1 (PAI-1) 4G > 5G promoter rs1799889 4G > 5G with stroke patients**									
**Subjects**	N=	5G	4G/5G	4G	Df	X^2^	5G	4G	p- value
**Cases**	100	24 (24%)	45 (45%)	31 (31%)	2	8.43	0.57	0.53	0.014
**Controls**	110	46 (41.81%)	43 (39%)	21 (19%)			0.62	0.38	

### Association between ANRIL-A > C, ANRIL-G > A, HNF1α G > T and PAI-1 4G > 5G genotypes with clinical charateristcs of stroke patients

We examined the correlation between ANRIL-A > C, ANRIL-G > A, HNF1Α G > T, and PAI-1 4G > 5G genotypes and laboratory parameters such as blood sugar, cholesterol, and LDL among stroke patients. Notably, stroke patients with the CA genotype of ANRIL-A > C, AA genotype of ANRIL-G > A, GT alleles of HNF1α, and 4G genotype of PAI-1 exhibited significantly higher levels of cholesterol and blood sugar when compared to individuals with other genotypes. Additionally, higher levels of LDL were observed in stroke patients carrying the GA and AA genotypes of ANRIL-G > A in comparison to those with the GG genotype, as illustrated in S1 Fig.

### Allelic genotypes of ANRIL, HNF1α and PAI-1 genes predict the risk of stroke

Our findings demonstrated a significant association between the lncRNA ANRIL rs1333048-GA genotype and susceptibility to stroke in the codominant model, with an odds ratio (OR) of 2.29 (95% CI = 1.2465 to 4.213), a relative risk (RR) of 1.54 (95% CI = 1.1090 to 2.149), and a p-value of less than 0.007 (Table 4). In the dominant inheritance model, a strong association was observed between the ANRIL-CC genotype and the combined (CA + AA) genotypes with susceptibility to stroke, yielding an OR of 1.83 (95% CI = 1.0456 to 3.214), RR of 1.35 (95% CI = 0.9811 to 1.579), and a p-value less than 0.034. Conversely, in the dominant inheritance model, a protective correlation was found between the ANRIL-CA genotype and the combined CC + AA genotypes with stroke susceptibility, resulting in an OR of 0.42 (95% CI = 0.239–0.765), RR of 0.64, and a p-value less than 0.0042 (Table 4). In allelic comparison, the ANRIL-A allele was not associated with susceptibility to stroke, as indicated by an OR of 1.25 (95% CI = 0.8260 to 1.911), RR of 1.12, and a p-value of 0.0001.

**Table 4:** Association of ANRIL rs1333048A>C genotypes with the Stroke risk.

Genotypes	Healthy controls	Stroke cases	OR (95% CI)	Risk Ratio(RR)	P-Value
**Codominant inheritance model**					
ANRIL -CC	55	40	1 (reference)	1 (reference)	
ANRIL -GA	30	50	2.29(1.2465 to 4.213)	1.54(1.1090 to 2.149)	0.007
ANRIL -AA	15	10	0.91(0.3735 to 2.2498)	0.96(0.6711 to 1.387)	0.84
**Dominant inheritance model**					
ANRIL -CC	55	40	1(reference)	1(reference)	
ANRIL -(CA + AA)	45	60	1.83(1.0456 to 3.214)	1.35(0.9811 to 1.579)	0.034
**Recessive inheritance model**					
ANRIL (CC + GA)	85	90	1(reference)	1(reference)	
ANRIL -AA	15	10	0.62(0.2682 to 1.478)	0.80(0.5679 to 1.154)	0.28
**Allele**					
ANRIL -C	140	130	1(reference)	1(reference)	
ANRIL -A	60	70	1.25 (0.8260 to 1.911)	1.12(0.9031 to 1.397)	0.28
**Over dominant inheritance model**					
ANRIL -CA	**30**	**50**	1(reference)	1(reference)	
ANRIL -CC + AA	**70**	**50**	0.42(0.2399 to 0.765)	0.64(0.4664 to 0.88)	0.0042

Our findings revealed a robust association between the ANRIL rs10757278 GG genotype and susceptibility to stroke in the codominant model, with an OR of 2.80 (95% CI = 1.3803 to 5.686), a RR of 1.66 (95% CI = 1.1611 to 2.388), and a p-value less than 0.004 (Table 5). In the recessive inheritance model, our study reported a significant association between the ANRIL genotypes (AA + AG) and susceptibility to stroke, with an OR of 3.14 (95% CI = 1.694 to 5.833), RR of 1.62 (95% CI = 1.1672 to 2.272), and a p-value less than 0.0003. In allelic comparison, the ANRIL-G allele exhibited a significant association with stroke susceptibility, as evidenced by an OR of 1.84 (95% CI = 1.2608 to 2.695), RR of 1.31 (1.108 to 1.570), and a p-value less than 0.0016. Our findings unveiled a significant association between the HNF1α-TT genotype and susceptibility to stroke in the codominant model, with an OR of 18.36 (95% CI = 1.0239 to 329.235), a RR of 9.96 (95% CI = 0.6267 to 139.376), and a p-value less than 0.048 (Table 5).

**Table 5:** Association of ANRIL rs10757278 G > A genotypes with the stroke risk.

Genotypes	Healthy controls	Stroke cases	OR (95% CI)	Risk Ratio(RR)	P-Value
**Codominant inheritance model**					
ANRIL -AA	41	24	**1 (reference)**	**1 (reference)**	
ANRIL -AG	54	35	1.10(0.5727 to 2.140)	1.03(0.8095 to 1.335)	0.76
ANRIL -GG	25	41	2.80(1.3803 to 5.686)	1.66(1.1611 to 2.388)	0.004
**Dominant inheritance model**					
ANRIL -AA	41	24	**1 (reference)**	**1 (reference)**	
ANRIL -(AG + GG)	79	76	1.64(0.9073 to 2.976)	1.23(0.9718 to 1.576)	0.101
**Recessive inheritance model**					
ANRIL (AA + AG)	95	59	**1 (reference)**	**1 (reference)**	
ANRIL -GG	25	41	3.14(1.6942 to 5.833)	1.62(1.1672 to 2.272)	0.0003
**Allele**					
ANRIL -A	136	83	**1 (reference)**	**1 (reference)**	
ANRIL -G	104	117	1.84 (1.2608 to 2.695)	1.31(1.1089 to 1.570)	0.0016
**Over dominant inheritance model**					
ANRIL -AG	54	35	**1 (reference)**	**1 (reference)**	
AA/GG	66	65	1.51(0.8801 to 2.623)	1.20(0.9488 to 1.528)	0.133

In the recessive inheritance model, a strong association was observed between the HNF1α (GG + GT) genotypes and the TT genotype with susceptibility to stroke, yielding an OR of 22.14 (95% CI = 1.261 to 388.66), RR of 10.18 (95% CI = 0.686 to 150.96), and a p-value less than 0.034 (Table 6). In allelic comparison, the HNF1α-T allele was notably associated with susceptibility to stroke, as indicated by an OR of 1.55 (95% CI = 1.0661 to 2.254), RR of 1.24, and a p-value of 0.021 (Table 6).

**Table 6:** Association of *HNF1α* (rs1169288) G > T (I27L) genotypes with the stroke risk.

Genotypes	Healthy controls	Stroke cases	OR (95% CI)	Risk Ratio(RR)	P-Value
**Codominant inheritance model**					
HNF1*α* -GG	40	37	**1(reference)**	**1(reference)**	
HNF1*α* -GT	80	55	0.74(0.4230 to 1.306)	0.87(0.6784 to 1.132)	0.314
HNF1*α* -TT	0	08	18.36(1.0239 to 329.235)	9.96(0.6267 to 139.376)	0.048
**Dominant inheritance model**					
*HNF1α* -GG	40	37	**1(reference)**	**1(reference)**	
*HNF1α* -(GT + TT)	80	63	0.85(0.4884 to 1.484)	0.92(0.7164 to 1.203)	**0.570**
**Recessive inheritance model**					
*HNF1α* (GG + GT)	120	92	**1(reference)**	**1(reference)**	
*HNF1α* -TT	0	8	22.14(1.2619 to 388.663)	10.18(0.686 to 150.966)	0.034
**Allele**					
HNF1*α* -G	160	129	**1(reference)**	**1(reference)**	
*HNF1α* -T	80	100	1.55 (1.0661 to 2.2546)	1.24(1.0266 to 1.511)	0.021
***Over dominant***					
HNF1*α* -GT	80	55	**1(reference)**	**1(reference)**	
HNF1*α* -GG + TT	40	45	1.63(0.9469 to 2.827)	1.25(0.9658 to 1.641)	0.08

### PAI-1 rs1799889 4G > 5G or PAI-1 4G > 5G promoter rs1799889 4G > 5G genotypes predicts the risk of stroke

A multivariate analysis using logistic regression, including OR or RR with 95% CI, was performed for each group to assess the relationship between PAI-1 rs1799889 4G > 5G genotypes and stroke risk (Table 7). In the codominant model, the PAI-1 -4G/5G genotype was associated with increased stroke risk, with an OR of 2.09 (95% CI = 1.0505 to 3.829), RR of 1.34 (1.0240 to 1.766), and a p-value of less than 0.034. A significant association was also found in the codominant model between the PAI-1-5G and PAI-1-4G genotypes, indicating increased stroke risk, with an OR of 2.00 (95% CI = 1.0505 to 3.829), RR of 1.62 (1.1228 to 2.358), and a p-value of less than 0.034. In the dominant inheritance model, a strong association was noted between the PAI-1-5G and PAI-1-(4G5G+4G) genotypes, reflecting enhanced stroke risk, with an OR of 2.27 (95% CI = 1.2552 to 4.127), RR of 1.43 (1.1224 to 1.841), and a p-value of less than 0.006. Similarly, a significant association was observed in the dominant inheritance model between the PAI-1-(5G + 4G/5G) and PAI-1-4G genotypes, indicating an increased stroke risk, with an OR of 1.90 (95% CI = 1.0071 to 3.600), RR of 1.39 (0.9754 to 1.994), and a p-value of less than 0.047. Furthermore, the 4G allele was protective against stroke risk, with an OR of 0.54 (95% CI = 0.3711 to 0.807), RR of 0.74 (0.6172 to 0.905), and a p-value of less than 0.0023.

**Table 7:** Association of PAI-1 4G > 5G promoter rs1799889 4G > 5G gene variation between stroke patients and controls.

Genotypes	Healthy Controls 100	Stroke Cases 110	OR (95% CI)	Risk Ratio(RR)	P-Value
**Codominant inheritance model**					
PAI-1-5G	46	24	**1 (reference)**	**1 (reference)**	
PAI-1-4G/5G	43	45	2.09(1.0505 to 3.829)	1.34(1.0240 to 1.766)	0.034
PAI-1-4G	21	31	2.00(1.0505 to 3.829)	1.62(1.1228 to 2.358)	0.034
**Dominant inheritance model**					
PAI-1-5G	46	24	**1 (reference)**	**1 (reference)**	
PAI-1-(4G5G+4G)	64	76	2.27(1.2552 to 4.127)	1.43(1.1224 to 1.841)	0.006
**Recessive inheritance model**					
PAI-1-(5G + 4G/5G)	89	69	**1 (reference)**	**1 (reference)**	
PAI-1-4G	21	31	1.90(1.0071 to 3.600)	1.39(0.9754 to 1.994)	0.047
**Allele**					
PAI-1-5G	85	107	**1 (reference)**	**1 (reference)**	
PAI-1-4G	135	93	0.54 (0.3711 to 0.807)	0.74(0.6172 to 0.905)	0.0023
**Over dominant**					
PAI-1-4G/5G	43	45	**1 (reference)**	**1 (reference)**	
PAI-1-5G + 4G	67	55	0.54(0.3711 to 0.807)	0.89(0.6810 to 1.162)	0.002

### Association between ANRIL-A > C, ANRIL-G > A, HNF1α G > T and PAI-1 4G > 5G genotypes with clinical charateristcs of stroke patients

**Fig 1:**
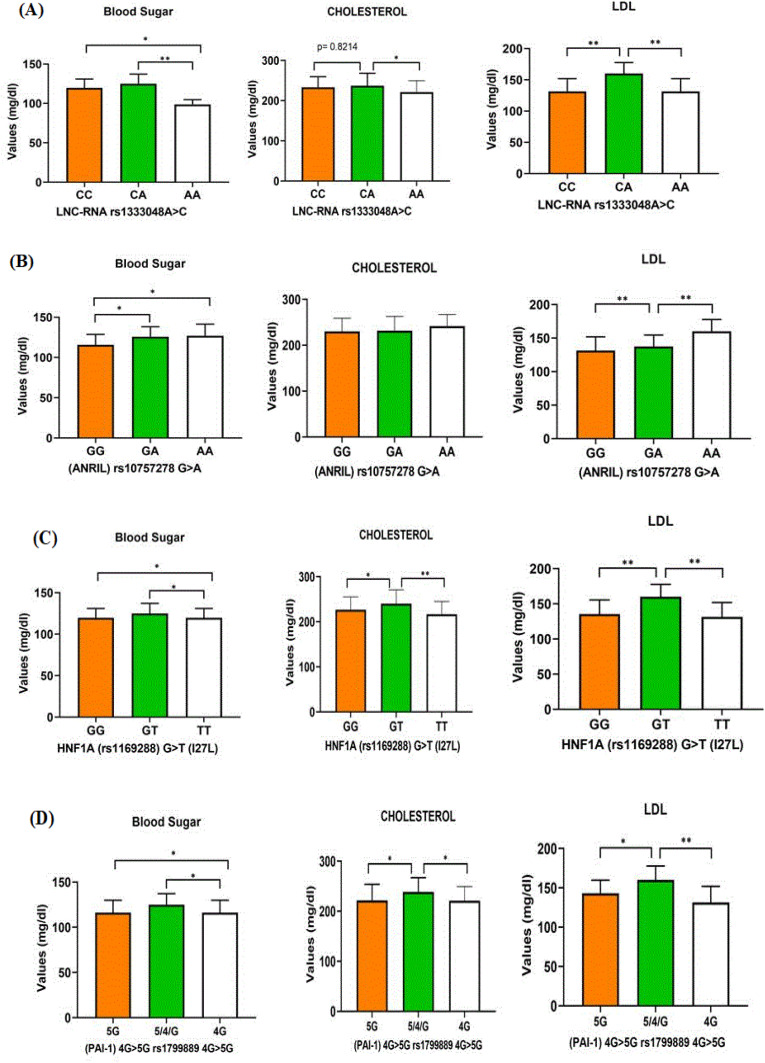
Clinical association of blood sugar, cholesterol and LDL in stroke patients. **(A)** Comparing the p value of Blood sugar, Cholesterol and LDL with ANRIL rs1333048A>C, **(B)** Comparing the p value of Blood sugar, Cholesterol and LDL with LNCRNA(ANRIL) rs10757278 G > A. **(C)** Comparing the p value of Blood sugar, Cholesterol and LDL with Hepatocyte nuclear factor-1 alpha HNF1Α (rs1169288) G > T. **(C)** Comparing the p value of Blood sugar, Cholesterol and LDL with Plasminogen activator inhibitor-1 (PAI-1) 4G > 5G promoter rs1799889 4G > 5G genotypes.

We examined the correlation between ANRIL-A > C, ANRIL-G > A, HNF1Α G > T, and PAI-1 4G > 5G genotypes and laboratory parameters such as blood sugar, cholesterol, and LDL among stroke patients. Notably, stroke patients with the CA genotype of ANRIL-A > C, AA genotype of ANRIL-G > A, GT alleles of HNF1α, and 4G genotype of PAI-1 exhibited significantly higher levels of cholesterol and blood sugar when compared to individuals with other genotypes. Additionally, higher levels of LDL were observed in stroke patients carrying the GA and AA genotypes of ANRIL-G > A in comparison to those with the GG genotype, as illustrated in Fig 1.

### Whole exome sequencing in stroke patients

The most prevalent COL4A2 gene variants, identified through WES in all our stroke patients, include COL4A2 rs4238272 (c.297G > A), COL4A2 rs439831 (c.3804T > A), COL4A2 rs409858 (c.3807T > C), and COL4A2 rs438758 (c.4083T > C) (Table 8). In the case of PSEN2 gene variants, the most common ones detected in all our stroke patients through WES are PSEN2 rs11405 (c.69T > C), PSEN2 rs6759 (c.129C > T), and PSEN2 rs1046240 (c.129C > T) (Table 8). For RNF213 gene variants, the predominant ones identified in all our stroke patients through whole exome sequencing are RNF213 rs8082521 (c.3544C > A), RNF213 rs8074015 (c.4139A > G), and RNF213 rs4890009 (c.4797G > A). Likewise, the most common NOTCH3 gene variants, as revealed by whole exome sequencing in all our stroke patients, include NOTCH3 rs15174241 (c.4563A > G), NOTCH3 rs15181626 (c.2742A > G), and NOTCH3 rs15192033 (c.606A > G) (Table 8).

**Table 8:** Rare and common gene variants identified in stroke patients by whole exome sequencing.

Gene	Chromosome	Variant type	Variant class	Base change	dpSNP_RS number	Exon number	Freq/10 samples	%
COL4A2	chr13	SNP	synonymous_variant	c.297G > A	rs4238272	(5/48)	10	100
COL4A2	chr13	SNP	synonymous_variant	c.3804T > A	rs439831	(41/48)	10	100
COL4A2	chr13	SNP	synonymous_variant	c.3807T > C	rs409858	(41/48)	10	100
COL4A2	chr13	SNP	synonymous_variant	c.4083T > C	rs438758	(43/48)	10	100
PSEN2	chr1	SNP	synonymous_variant	c.69T > C	rs11405	(4/15)	10	100
PSEN2	chr1	SNP	synonymous_variant	c.129C > T	rs6759	(4/15)	10	100
PSEN2	chr1	SNP	synonymous_variant	c.261C > T	rs1046240	(5/15)	10	100
RNF213	chr17	SNP	missense_variant	c.3544C > A	rs8082521	(21/69)	10	100
RNF213	chr17	SNP	missense_variant	c.4139A > G	rs8074015	(22/69)	10	100
RNF213	chr17	SNP	synonymous_variant	c.4797G > A	rs4890009	(25/69)	10	100
NOTCH3	chr19	SNP	synonymous_variant	c.4563A > G	rs15174241	(25/33)	10	100
NOTCH3	chr19	SNP	synonymous_variant	c.2742A > G	rs15181626	(17/33)	10	100
NOTCH3	chr19	SNP	synonymous_variant	c.606A > G	rs15192033	(4/33)	10	100

## Discussion

Stroke ranks as the second-leading global cause of death. Disturbingly, there has been a notable surge in new stroke cases (70%), existing stroke cases (85%), and related fatalities (43%) over the past decade [38]. Numerous risk factors, such as high blood pressure, diabetes, smoking, inactivity, and age and gender, significantly increase the likelihood of stroke [6]. Gender plays a significant role in stroke risk, as IS is more commonly observed in younger men and tends to become more prevalent in older women [39]. In contrast to earlier reports, which indicated that the age associated with IS risk was approximately 69.2 years in 2005, recent evidence suggests a shifting trend toward younger age groups [40]. Consistent with this evolving trend, our study presents data indicating a mean age of 58 years among stroke patients.

Polymorphic variations in genes have been the subject of extensive global studies, contributing valuable insights into various health conditions, including stroke. These variations playas crucial roles in the susceptibility and outcomes of stroke patients. Clinically, this is a significant endeavor since the identification of genetic markers that can predict the risk of stroke and coronary disease holds the potential to target individuals at risk for intensive risk modification or drug therapy. However, despite the wealth of research globally, there has been a notable dearth of such studies in KSA. Remarkably, our study addresses this gap as the first to investigate the genotype distribution in relation to gender and age among stroke patients in KSA. Our research focuses on four key genes (HNF1α, PAI1, lnc-RNA rs1333048 A > C, and ANRIL rs10757278 G > A), shedding light on polymorphic variations within this specific population. This groundbreaking work not only adds to the global understanding of stroke but also addresses the unique challenges and genetic factors within the context of Saudi Arabia, where stroke is a critical health concern.

Comprehensive evaluations are currently in progress to assess the therapeutic potential of lncRNAs in a wide range of medical conditions, including cardiovascular disorders [41]. In this connection, specific variations in the ANRIL gene within the INK4 locus have been proposed as potential contributors to stroke onset [21]. Our data analysis revealed higher cholesterol levels in patients with the lncRNA rs1333048 polymorphism. However, we observed a significant difference between the CA and AA alleles, leading us to conclude there’s no direct correlation with stroke risk. Previous research, like Na Bai’s work, emphasized a strong link between ANRIL and IS risk in Asian populations [42]. Future investigations could use Mendelian randomization analysis to clarify the roles of SNPs in ANRIL associated with IS [42]. Regarding the ANRIL SNP rs10757278, our findings showed a slight increase in cholesterol levels among patients with this polymorphism. Notably, both SNPs, rs1333048 and rs10757278, were connected to higher blood sugar and LDL levels in these patients, indicating a robust link with stroke and highlighting their potential as cardiovascular disease risk factors [17]. Xuemei Han and colleagues identified rs10757278 as a risk locus for IS, despite its protective effects in certain contexts, emphasizing the growing significance of genetic biomarker research in understanding disease susceptibility [43].

Genetic variations within the HNF-1α gene, which exert pleiotropic effects by influencing multiple causative pathways, offer compelling prospects for connections with intricate, multifactorial vascular conditions, such as stroke [44]. Zhou, YJ et al showed that HNF1α SNPs are associated with elevated blood ApoA1 levels [45]. These findings suggest that polymorphic variations in the HNF1α locus may indeed serve as risk factors for both coronary artery disease (CAD) and IS [45]. These findings are supported by our study, which shows that polymorphic variations in the HNF1α locus may possibly raise the risk of IS. Our results also show a strong association between the HNF1α SNP, rs1169288 Ile27Leu, and elevated levels of cholesterol and LDL. It is important to note that the connection between this SNP, serum lipid traits, and its impact on stroke risk had not been established previously [26]. Our findings also reveal increased blood sugar levels in patients with the same SNP. In summary, our findings align with existing studies, collectively suggesting the potential involvement of HNF-1α SNPs in elevating the risk of stroke. This research contributes to the expanding body of evidence underscoring the significance of HNF-1α SNPs in susceptibility to stroke.

Within the domain of stroke risk factors, some researchers have emphasized the protective role attributed to the 4G/4G genotype of the PAI-1 gene [34]. In a meta-analysis conducted by Mohammad Ali Jafari and colleagues, they convincingly demonstrated that the PAI-1 rs1799889 polymorphism significantly heightens the risk of IS, especially among Asian populations [46]. In contrast, our own data revealed that individuals carrying the PAI-1 4G/5G polymorphism displayed elevated levels of blood sugar, cholesterol, and LDL, implying a potentially significant role in stroke risk. Furthermore, a study conducted by Liu, et al, delved into the functions of PAI-1 gene polymorphisms within the context of atherosclerotic diseases. Their findings suggested that specific polymorphisms, including rs1799889, could serve as potential genetic biomarkers for atherosclerotic conditions [26]. These contrasting findings underscore the intricate and multifaceted genetic contributions to stroke risk, underscoring the ongoing need for extensive research in this field.

Recent technological advancements, notably the availability of WES, have opened up new opportunities for comprehensive investigations of multiple genes [47]. This approach allows for the development of gene panels that assist in interpreting exome sequencing results related to monogenic stroke. These gene panels can be invaluable in evaluating the pathogenicity of novel variants within these genes, as exemplified by the study conducted by Hartl et al, [48]. Their study highlighted the pathogenic nature of the NOTCH3 c.1672C > T (p.Arg558Cys) genotype, associated with cerebral autosomal dominant arteriopathy with subcortical infarcts and leukoencephalopathy [48]. Interestingly, the same individual was found to carry a previously unreported COL4A1 c.161C > T (p.Pro54Leu) variant, which appeared to segregate with the disease within their family and was also considered pathogenic. Xiaoling Yuan et al, [49] reported on the association of NOTCH3 381C > T and 1735T > C gene variants with stroke, suggesting their role as risk factors in stroke development [49]. However, the NOTCH3 605C > T polymorphism did not exhibit the same association. In concordance with these studies, we identified three of the most common gene variants through WES in the NOTCH3 gene: NOTCH3 rs15174241 (c.4563A > G), rs15181626 (c.2742A > G), and rs15192033 (c.606A > G) (Table 8). Similarly, mutations in presenilin 1 (PSEN1) on chromosome 14, presenilin 2 (PSEN2) on chromosome 1, and Amyloid precursor protein (APP) on chromosome 21 genes have been linked to most early-onset, autosomal dominant Alzheimer’s disease [50]. Identification of pathogenic mutations in the PSEN2 gene in a Korean patient with early-onset Alzheimer’s disease emphasizes the significance of PSEN2 mutations [51]. In concordinance with these earier studies, we discovered three prevalent PSEN2 gene variants, PSEN2 rs11405 (c.69T > C), rs6759 (c.129C > T), and rs1046240 (c.129C > T) in our stroke patient cohort (Table 8). As per previous strudies highlighting the pathogenicity of the COL4A2 p.Glu1123Gly variant [52], our research also identified four of the most prevalent COL4A2 gene variants in our stroke patients: COL4A2 rs4238272 (c.297G > A), rs439831 (c.3804T > A), rs409858 (c.3807T > C), and rs438758 (c.4083T > C) (Table 8).

Teppei Kamimura and colleagues’ study shed light on the importance of the RNF213 p.R4810K variant in early-onset IS, particularly in the context of anterior circulation stenosis in Japan [53]. This variant was more prevalent in women than men and was associated with vasculogenesis rather than atherogenesis. Patients with this variant were predisposed to small intracranial arteries, potentially leading to hemodynamic compromise in the presence of intracranial atherosclerosis [54]. Similrly, we have identified several of the most common RNF213 gene variants through WES in our stroke patients: RNF213 rs8082521 (c.3544C > A), rs8074015 (c.4139A > G), and rs4890009 (c.4797G > A) (Table 8). In conclusion, the application of whole-exome sequencing has significantly expanded our capacity to explore and understand the genetic underpinnings of stroke. These advances have revealed potential pathogenic variants in genes associated with stroke and other neurological conditions. Such insights contribute to our growing understanding of the genetic factors involved in stroke susceptibility and pave the way for targeted approaches to diagnosis and treatment.

### Conclusion

In conclusion, our study provides persuasive evidence that particular genetic factors found within the lncRNA on the ANRIL locus, including the HNF1A-A > C (p.I27L) variant and the PAI-14G > 5G gene, are associated with a higher risk of stroke. This study has uncovered low, novel, and intermediate-genetic-risk variants within COL4A2, PSEN2, NOTCH3, and RNF213 through WES. To deepen our comprehension and validate these findings, further investigations in larger cohort studies are essential. Additionally, conducting functional analyses to assess the causal effects of these genetic variants on specific stroke subtypes is of paramount importance. Our study lays the groundwork for ongoing exploration of the genetic factors contributing to stroke susceptibility and its various subtypes. These findings require further verification in large scale case-control and protein functional studies before being used for genetic testing for prevention and treatment of stroke. Our study lays the groundwork for ongoing exploration of the genetic factors contributing to stroke susceptibility and its various subtypes.

Limitations of this study include a relatively small sample size in one population, a cross-sectional design, and the imbalanced age distribution between cases and controls. Further protein functional studies and large-scale longitudinal case-control studies considering environmental factors (e.g., diet and physical activity) in different populations are required to validate these findings before these SNVs considered for genetic testing.

## Supporting information

S1 Fig Association between ANRIL-A > C (A), ANRIL-G > A (B), HNF1α G > T (C) and PAI-1 4G > 5G (D) genotypes with clinical charateristcs of stroke patients. (DOCX)
