# Supplementary material for: Exploring the genetic frontier: Unraveling ANRIL, PAI-1, and HNF1α in stroke progression
Source: PLoS One. 2025 Dec 11;20(12):e0332252. doi: 10.1371/journal.pone.0332252 (PMC12698005; doi:10.1371/journal.pone.0332252)
Supplement: S1 Fig — (DOCX) [file pone.0332252.s001.docx]

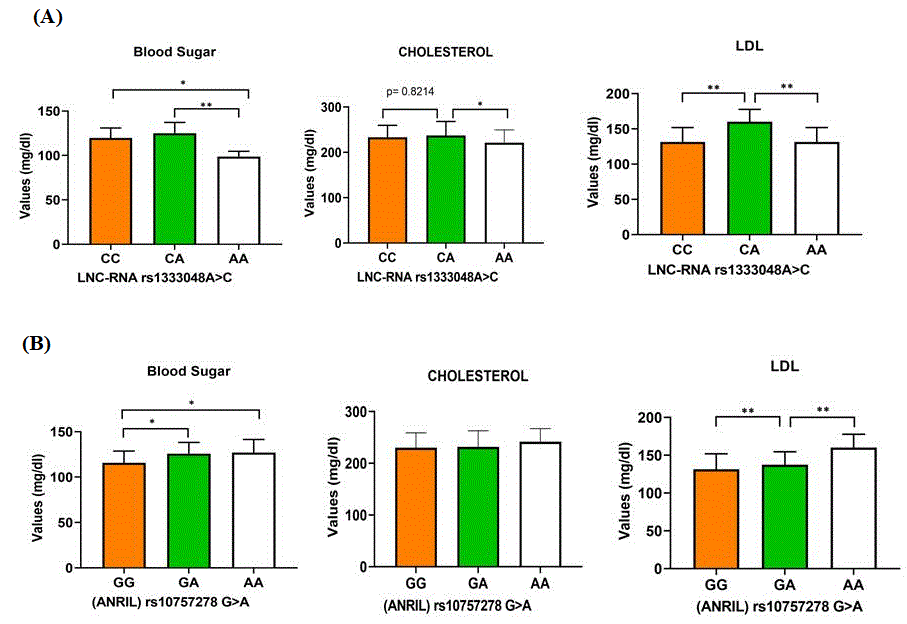

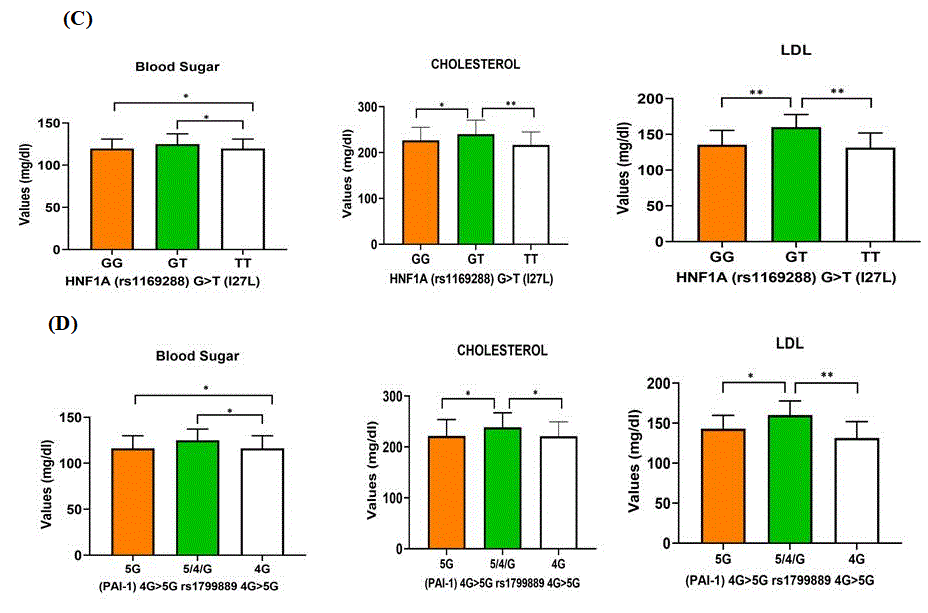


**S1 Fig**. Association between ANRIL-A>C **(A),** ANRIL-G>A **(B)**, HNF1α G>T **(C**) and PAI-1 4G>5G **(D)** genotypes with clinical charateristcs of stroke patients.
